# Whither Are We Drifting?

**Published:** 1889-12

**Authors:** 


### WHITHER ARE WE DRIFTING?

In the course of a sermon on the evangelization of New York, the Rev. Heber Newton said: "The churches have been converted into the paganism of political economy. Unless the clergy look sharp they will find they have accepted retainers from capital, and their lips are silenced when labor piteously cries for justice. No word against the root evils of our industrial system is spoken from hosts of the pulpits, whence the carpenter's son has been driven by Mammon. The poor man hears the gospel of "property, property, property!" Charity is preached, but not justice and it is not charity but justice that the world needs. If the ethical forces of the church were turned on these problems some solution would soon be found. The failure to find a solution is a terrible indictment of Christianity. As a class the very poor are as irreligious as the very rich, and we are gravitating into a city of the extremes. We have two dangerous classes to contend with. Is it any wonder that religion seems disappearing in the gulf where a prosperous middle class has gone down? If we can convert the rich I have no fears about the poor. When their big brothers on earth are brotherly, they will believe in a Father in Heaven. It is high time for our rich men to remember that when the masses lose respect for religion there will be small security for wealth. Property, then, had better be put again into diamonds. Real estate in Sodom is liable to a sudden shrinkage.

